# Perinatal mental health care from the user and provider perspective: protocol for a qualitative study in Switzerland

**DOI:** 10.1186/s12978-020-0882-7

**Published:** 2020-02-17

**Authors:** Anke Berger, Karin Schenk, Ankica Ging, Sebastian Walther, Eva Cignacco

**Affiliations:** 1 Department of Health Professions, Bern University of Applied Sciences, Bern, Switzerland; 2 University Hospital of Psychiatry, University of Bern, Bern, Switzerland; 3 Translational Research Center, University Hospital of Psychiatry, University of Bern, Bern, Switzerland

**Keywords:** Perinatal, Mental disorder, Health care, Access to health care, User perspective, Provider perspective, Qualitative study

## Abstract

**Background:**

Mental disorders in the perinatal period (PMD) can severely harm women and their children if not detected early and treated appropriately. Even though mental health care is covered by health insurance and is used widely by women in the perinatal period in Switzerland, it is not known if the care provided is meeting the needs of the patients and is efficient in the view of health care professionals. The aim of this study is to identify strengths, gaps and requirements for adequate mental health care in the perinatal period from the perspectives of patients and care providers for a wide range of relevant mental disorders.

**Methods:**

In the qualitative study we conduct (1) semi-structured single interviews with former PMD patients to obtain narratives about their experiences and needs for health care for their condition. Women are included who have been treated for PMD but are mentally stable at the time of the interview (*n* = 24). We will stratify the sample by 4 clusters of relevant ICD-10 F-diagnoses, covering the most frequent and the most severe mental disorders. We will further stratify the sample based on whether the women already had experience with psychiatric or psychological health care or not before their last episode of PMD. We will also conduct (2) three interprofessional focus groups with health and social care professionals involved in perinatal care, and a health insurance representative. The focus groups will consist of 5–8 professionals. Data collection and thematic analysis will consider Levesque’s et al. (2013) conceptual model on access to health care.

**Discussion:**

The study will provide fundamental data on the experiences and perspectives about perinatal mental health care from user and provider perspectives. The study will generate the evidence base needed to develop models of integrated, coordinated, patient- and family-centred care that is accessed by women with various types of PMD.

**Trial registration:**

The study was registered on ClinicalTrials.gov in November 2019 under the identifier NCT04185896.

## Plain English summary

### Background

Up to one in five women may have a mental disorder during pregnancy or in the first year after delivery. It is crucial to identify and treat these women early and appropriately to avoid adverse consequences like birth complications, persistent mental illness or child development problems. In Switzerland, many pregnant or postnatal women receive mental health care, but we do not know whether the health care is timely, accessible and appropriate. The aim of this study is to identify the requirements for adequate mental health care during pregnancy and after childbirth.

### Methods

We conduct individual interviews with women about their experiences with health care during pregnancy or after childbirth. We interview women with different mental disorders to cover a broad range of needs toward health care. We take into account whether the women had experience with mental health care from earlier phases of life. We also conduct group interviews with health and social care professionals. We categorize the statements of the interviewees into topics that are relevant to health care provision of affected women. An established theory about access to health care is considered in the analysis.

### Discussion

The study will provide fundamental information to improve health care to women with mental health problems during pregnancy and after birth. The conclusions of the study will help to improve mental health care for this vulnerable population.

## Background

### Prevalence and consequences of perinatal mental disorders

Perinatal mental disorders (PMD) are mental and behavioural disorders that occur during pregnancy or in the first year postpartum. PMD may affect up to 20% of women [1, 2] and can severely harm women and their children if not detected and treated appropriately. For example, women suffering from PMD have poorer obstetric outcomes [3, 4] and severe PMD are associated with a 70-fold increased suicide risk in the first year post-partum [5]. In an Australian study, suicide and accidental injury accounted for 62% of deaths of women in this period and injuries categorized as accidental might be intentional [6]. Children of mothers with PMD are at risk of early psychopathology and persistent emotional, behavioural, or cognitive problems [4, 7]. In-adequate treatment of PMD were estimated to cost the UK about £8.1 billion in direct and indirect costs – almost £10′000 per birth [8]. Most of the costs (72%) came from the adverse consequences of PMD for children. The potential of PMD to severely harm women and their children and high follow-up costs call for specific interventions to facilitate service utilization and improve mental health care for this vulnerable group.

### Mental health care in the perinatal period

#### Supply and use of mental health care

In high-income-countries like Switzerland, general mental health services usually are abundant, covered by health insurance, and most services can be accessed directly [9]. Nevertheless, more than 50% of people in the general population with mental disorders remain untreated [10, 11]. In the perinatal population of women, rates of undiagnosed women might be even higher [12, 13] and treatment rates may be significantly lower than in non-pregnant cohorts [12, 14].

In a population based study we found that 16.7% of perinatal women per year used mental health services at least once [15]. Women with PMD seemed to prefer out-patient settings and the most frequent treatment for PMD was psychotropic medication. Up to 10% of women had used mental health care already before pregnancy [15]. The fact that half of these women stopped using mental health services during pregnancy is concerning because they had no professional support to help them adjust to the emotional, physical and social challenges of the perinatal period. Only 1% of perinatal women received mental health care in obstetric hospitals. Freelance midwives visiting women at home during post-partum care recorded PMD rarely, too [15]. It appears that there are significant health care gaps and the care provided might not be timely, adequate, or efficient.

#### Barriers to appropriate health care

Barriers that prevent perinatal women from accessing mental health services are complex and interlinking [16]. Specific barriers include stigma related to mental illness [11, 17–19]. Women do not seek help because they feel ashamed or embarrassed to admit to mental illness in this period of life [13, 18]. Women may avoid mental health services because mothers with severe mental disorders are seen as incapable of caring for their children [20]. Among British women who experienced postpartum depression, 30% reported that they never spoke to health professionals about their symptoms [21]. The acceptability of pharmacotherapy may be lower during pregnancy and breastfeeding, and when psychotherapy is available, it may not be accessible quickly enough [12, 13, 17]. Lack of affordable childcare may pose another barrier [13]. Some women normalize their symptoms and misunderstand them as an adjustment to motherhood, or may not report them because they are unfamiliar with mental illness and possible treatments [13, 20]. Similar barriers might exist in the social environment of women with PMD. Friends and family might tend do normalize symptoms and may not support women who seek help [18]. Some women report that their family, friends, or partners expect women to be happy in this period of life [18].

On the provider side, systematic screening for PMD is missing, and structural or procedural barriers in the health care system prevail [19, 22, 23]. For example, Swiss psychiatric and gynaecological societies have not yet developed guidelines for PMD care. We could not find guidelines from cantons, hospitals or professional associations via search engines or literature databases. Specialized inpatient care in obstetric hospitals is rare in Switzerland [9] and obstetricians and midwives, who provide most of the care for perinatal women in Switzerland, rarely feel sufficiently trained to deal with women at risk or with symptoms of PMD [24, 25]. Women might need seamless transitions and good communication between services or units, but models of care can be disconnected [8, 13, 19, 22]. For example, when women are screened in obstetric care, an acceptable and timely intervention may not be available in the same setting [13]. Personnel in the obstetric setting are not equipped to deal with mental illness [24, 26, 27], and may not know about pharmaceuticals that do not harm the embryo, foetus, or new-born. Continued support can only be guaranteed when multidisciplinary perinatal services include expertise in maternity services and offer mental health treatment within the unit.

### Theoretical framework

Barriers to access to health care significantly contribute to in-adequate care. Levesque et al. [28] define access to health care as “the opportunity to reach and obtain appropriate health care services in situations of perceived need for care”. Access is determined by the intersection of personal (user) and institutional (provider) traits that can interfere with or support the “pathway of utilisation”. They define five consecutive steps to gain access to care; in the context of PMD these might be 1. the need for PMD treatment (e.g. risk factors, vulnerability to PMD; symptoms of PMD), 2. the ability to recognize health care needs and desired care, 3. seeking care for PMD, 4. reaching and obtaining PMD care; and, 5. receiving health care services appropriate to the needs of individual patients with PMD. The provider side contributes to the interface supply-related determinants of accessibility, i.e. approachability, acceptability, availability and accommodation, affordability and appropriateness. On the user side the abilities to perceive, seek, reach, pay for, and engage with health care services prevail [28]. We use this framework to capture experiences and needs of former PMD patients and health professionals with regards to access to perinatal mental health care.

### Research gaps

Mental disorders in the perinatal phase are widespread and can cause serious harm to women, their new-borns and families. In Switzerland mental health care is covered by mandatory insurance and many perinatal women use mental health care, mainly in non-obstetric outpatient settings where they are treated by mental health specialists (psychiatrists, psychologists) and primary care physicians [15]. It is not clear if this care meets the needs of affected women, or if all women have full access, as defined by Levesque et al. [28]. Studies have usually explored the needs of patients with postpartum depression [21, 29–33], but not the possibly different needs of women with other common or severe PMD like anxiety disorders, drug abuse disorders, and psychotic disorders. Since PMD carries a stigma [11, 34], many women may not access health services and go untreated. Specialized care for PMD is complex. It must be easily accessible and patient-oriented. Women need to be able to make informed decisions about suitable treatment and consider benefits and adverse effects of medication vs. psychotherapy. Inpatient psychiatric care has to respect bonding and attachment processes.

There is so far little information on womens’ needs and preferences for PMD health care and treatment. Nor do we know the views of professionals involved in perinatal health care what is required to adequately assess and treat affected women.

### Objectives

1. Identify the experiences and needs of PMD patients regarding perinatal mental health care
2. Capture the perspectives of health professionals and social care providers who care for perinatal women, their children and families on perinatal mental health care
3. Describe facilitators and barriers to perinatal mental health care provision based on a conceptual model of access to care
4. Extend the previous research focus on postnatal depression to include a wide range of common and severe PMDs so the diverse needs of women with different PMD can be captured.

## Methods/design

### Aim of the study

The aim of this study is to gain basic insights into the strengths, gaps and requirements for adequate mental health care in the perinatal phase in a high-income country. The study aims to gain a broad understanding of the problem by combining the perspectives of both patients and care providers for a wide range of relevant mental disorders defined by the ICD-10 diagnoses.

### Study design and setting

The study takes a qualitative explorative approach. It uses single semi-structured interviews to capture experiences and needs of 24 former PMD patients (part 1, user perspective). We will conduct three focus group interviews based on a semi-structured interview guide to gather experiences and perspectives of health professionals and social care providers on perinatal health care for patients with PMD (part 2, provider perspective).

The study population of part 1 will be former patients of a specialized gynecological-psychiatric consultation service offered at the Psychiatric University Hospital of Bern, Switzerland. Health and social care professionals for part 2 will be recruited in Bern and in close-by cantons of Aarau, Solothurn and Basel.

### Outcomes

Outcomes are narratives of experiences and perspectives of former PMD patients and health and social care professionals. Further outcomes will be descriptions of relevant barriers and facilitators to perinatal mental health care. We also will describe recommendations for perinatal mental health care.

As baseline characteristics of the sample of part 1 we will assess quantitatively 1. current psychological burden by the Brief Symptom Inventory [35] and 2. socio-demographic parameters (supplementary Table 2, Additional file 1). As baseline characteristics of the focus group members we will assess their socio-demographic parameters (supplementary Table 4, Additional file 1).

### Sample

The study sample for part 1 consists in women with previously experienced PMD. The sample for part 2 are health professionals and social care providers working with perinatal women and having dealt with women with PMD, and a health insurance delegate.

#### Women with former PMD

**Fig. 1:**
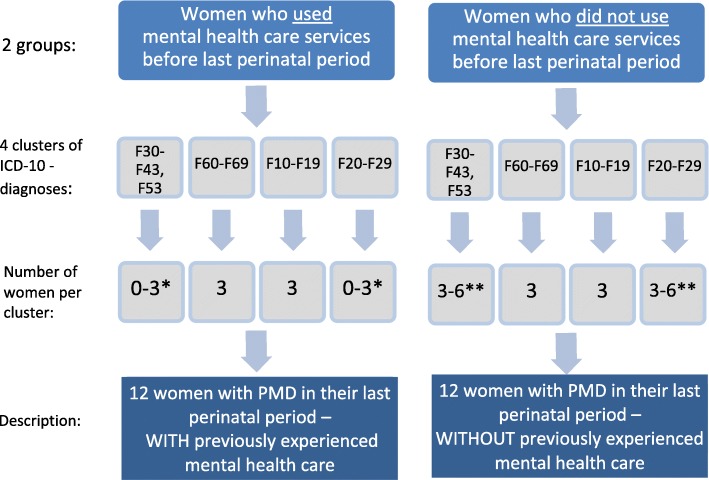
Inclusion chart: 24 women with perinatal mental disorder (PMD) in their past perinatal period will be recruited for the study. All included women are mentally stable for at least 12 months before the interview. Women who are pregnant again are excluded. For further inclusion or exclusion criteria see text. *Number of women in these clusters may range between 0 and 3 because of possible difficulties in recruiting women with these F-diagnoses who had experienced mental health care before pregnancy. **If the number of recruited women with previous mental health care experience in this PMD - cluster is less than 3, we will recruit more women without previous experience in mental health care to reach a total number of six women per cluster of ICD-10 diagnoses

The sample size of women with former PMD will be *n* = 24 (Fig. 1). This should be large enough to achieve data saturation in the interviews. Data saturation is achieved when no additional new information is gathered in additional interviews. If data saturation would not be achieved with 24 interviews, we would continue sampling and conduct further interviews to achieve data saturation.

##### Eligibility criteria

We will include women with a history of PMD who meet the following criteria:

- Have had PMD within the past 24 months before the interview
- Have recovered from acute PMD and are stable at least 12 months before the interview;
- Are considered able to share their experiences;
- Are more than 18 years old
- Speak and understand German.
- Have received PMD diagnoses from one of the following 4 groups of psychiatric disorders (ICD-10 Chapter V, F00-F99) [36]:
  1. Mood (affective) disorders (F30-F39); Anxiety disorders (F41); Reaction to severe stress, and adjustment disorders (F43); and Obsessive-compulsive disorder (F42);
  2. Disorders of adult personality and behavior (F60-F69; especially F60.3 Emotionally unstable personality disorder);
  3. Mental and behavioral disorders due to psychoactive substance use (F10-F19; excluding tobacco (F17) if it is the sole F-diagnosis of the patient);
  4. Psychosis (F20-F29 Schizophrenia, schizotypal and delusional disorders).

Women will be grouped into 4 clusters of psychiatric diagnoses (Fig. 1). The first cluster represents the most frequent types of PMD, which are often co-morbid. Diagnoses from cluster 2 are also frequent in the perinatal period. Cluster 3 and 4 represent severe PMD requiring different care. We will try to include six women to each cluster of PMD; three of these women will have already received mental health care before their last pregnancy, and three of these women will not have received mental health care before their last pregnancy. We define received mental health care as any care that was provided by a psychiatrist or by a psychological therapist.

##### Exclusion criteria

We will exclude women who are pregnant because physiological adaptations to pregnancy might influence the emotional and mental well-being.

#### Health professionals and social care providers

For study part 2 we will conduct three interprofessional focus groups with 6–8 participants each (total *n* = 18–24). A minimum of 3 focus groups is needed to obtain representative information [37]. We assume that our sample size is large enough to achieve data saturation. If data saturation would not be achieved after conducting the three focus groups, we would conduct further focus group interviews to achieve data saturation.

We will sample health care professionals and social care providers who work with perinatal women in in- and outpatient settings, and a health insurance delegate. Care providers include gynecologists, medical primary care providers, pediatricians, neonatologists, psychiatrists, psychologists, nurses, midwives, social care providers (community and hospital service) and community care nurses.

##### Eligibility criteria

Health professionals and social care providers will be included if they

- Have professional experience of at least 2 years,
- Have cared for at least two PMD patients the year before the focus group, and
- Understand and speak German.

### Recruitment

#### Women with former PMD

Our study partners from the Bern University Hospital of Psychiatry will support us in consecutively recruiting 24 women with former PMD. They will identify former patients from their database and contact women who meet the above inclusion criteria to ask them if they would be interested in participating in the study and specifically in a personal interview of about 2 h (including framing, interview and filling in a short questionnaire to assess their socio-economic background and the Brief Symptom Inventory (BSI) [35] at any location the women feels convenient with. If a woman is interested in participating, our study partners will ask her to agree in writing that the study partner can forward her contact information and diagnosis to the study coordinators at the Bern University of Applied Sciences. If the woman agrees to have her contact information forwarded to the study coordinators, the local study partners will provide us with her contact data and previous PMD ICD10-F-diagnosis. Afterwards, the study team will give the interested woman standardized written information describing the purpose and benefits of the study, and the time and effort required to participate. A member of the project team will also contact her by phone, answer any open questions and, if the woman is still interested, make an appointment for the interview. The woman may choose to be interviewed at home, in a conference room at Bern University of Applied Sciences, or in another place she prefers. If a woman decides not to participate in the study, we will delete all the information we received about her.

#### Health professionals and social care providers

We will recruit health professionals and social care providers in Bern, Aarau and Basel through professional associations, communities, and professional networks of the study team. The study team will contact potential participants with written standardized information about the purpose and benefits of the study and the time and effort participation will require. Health professionals and social care providers will be invited to participate in one of three focus groups conducted in Bern.

We will analyze the first 3–4 individual interviews with women ad interim analysis and will use any relevant information to update the focus group semi-structured interview guide.

### Data collection

#### Individual interviews with women who had PMD

We will conduct semi-structured individual interviews (INI) to capture the personal experiences and needs of women who suffered from PMD. We will use a semi-structured interview guide which will include open-ended questions informed by the literature, experience of the study team and a test run with a previous PMD patient in our net works. The interview guide will be framed by the conceptual model of access to health care by Levesque et al. [28]. We will pilot the guide with the first two women in the study and revise it for further interviews. The interviews will be conducted by trained health professionals (nurse, psychologist or midwife) and will be audio-recorded for transcription and analysis. The interviewer first informs the participant about the purpose and benefits of the study and obtains written informed consent. The participants next will fill in the BSI [35] to assess psychological distress during the past 7 days before the interview. Then the semi-structured interview will be conducted (supplementary Table 1, Additional file 1). After the interview, participants will be asked to complete a survey to collect socio-demographic data that describes women’s characteristic (supplementary Table 2, Additional file 1). Welcome and introduction to the interview, filling in questionnaires and information at the end of the interview will last 60–90 min, depending on the length of the narrative of the participant.

#### Focus groups with professionals

We will conduct focus groups with health professionals, social care providers, and delegates of a health insurance company to gather rich information about their range of experiences and perspectives. Our study has an interdisciplinary focus, so we will ensure a mix of participants from all relevant disciplines. Interactive discussions among participants should reveal similarities and differences in the experiences of participants. Two members of the research group will guide the focus groups. One person will moderate the interview and one person will ensure the groups run smoothly. Before the focus group interview, the moderator will inform the participants about the purpose and procedure of the focus group interviews. When written informed consent is obtained of all participants, the focus group interview will begin based on a semi-structured questionnaire. It will include questions informed by the literature, and draw on the conceptual model of access to care of Levesque et al. [28], as well as on the expertise of the research team. We will ask interview questions in the style of Krueger and Casey [37]. We will continually update the interview guide for the focus groups, based on findings from our individual interviews with former PMD patients so we can generate user-driven questions [38]. The interview guide for the focus group will also be adapted, based on experiences with the first focus group interview. The focus groups will last about 90 min, depending on the length of the contributions of the participants. A draft of the interview guide is provided (supplementary Table 3, Additional file 1).

#### Withdrawal and discontinuation

Participants of the semi-structured interviews and the focus groups will be informed about their opportunity to withdraw from informed consent at any time and without any consequences. All collected personal information will be deleted if a participant withdraws consent to participate at any point during the study.

#### Data recording and source data

The semi-structured interviews with former PMD patients and the focus group interviews with health professionals will be audio-recorded and transcribed verbatim. We will use the software program F4transkript (f4transkript Version 6.2.4 Edu, dr.dresing & pehl GmbH, Marburg, Germany) for the transcription of the recordings. Data will be analyzed using ATLAS.ti software (ATLAS.ti Scientific Software Development GmbH, Germany). Data collected with the questionnaires (demographic data and BSI) will be transferred to SPSS (IBM SPSS Statistics Version 23.0, IBM Corp, Armonk, NY, USA) for descriptive analyses of the data. Data entry will be double checked.

No subject identifiers other than the subject’s assigned unique study identifier will be contained in any of the source data or in any of the data files, which will be stored in the locked secure research servers or archive of the research team of the University of Applied Sciences, Department of Health Professions.

### Data analysis

The content of the interviews will be independently analyzed by 2–3 members of the research team, using thematic analysis [39]. The codes are compared in joint sessions to seek consensus. Thematic analysis first summarizes the transcribed original text by dividing the text into small units (paragraphs, sentences or phrases) that will then be categorized. The categories will describe the content of the interviews. During the analysis, members of the research team will be blinded to individual data of participants in the interviews and focus groups. The research team will discuss the categories until they reach consensus. Primary subcodes will be combined to superior codes and these will be grouped to themes. Data will be analyzed using ATLAS.ti software (ATLAS.ti Scientific Software Development GmbH, Germany).

### Ethical considerations

The study procedure includes single interviews with formerly PMD affected women. A sensitive approach will be taken to addressing and interviewing women who have suffered from PMD, since they are potentially vulnerable. For ethical reasons, we will interview only women who have recovered from acute PMD and are stable for at least 12 months before the interview.

If stressful emotions arise during interviews because women recall negative memories [40], we will offer individualized psychological support, provided by a professional contact person. If this professional considers follow-up psychological support is necessary, the woman will be referred to mental health care without delay. Interviewers will be health professionals (nurse, psychologist or midwife) who will be trained to perform the interviews, and to recognize patterns of burden by the study partners from psychiatry. All participants will be informed that their contribution to this study is optional, and that they have the right to withdraw their consent at any time. The anonymity of all information collected through interviews and questionnaires is assured by coding and sufficient data aggregation.

Due to the subject of our study, stressful emotions may also arise in participants of the focus groups or in the interviewers. Participants of the focus group as well as research assistants will have the opportunity to contact a member of the study team to talk about it. Within the research team, in regular meetings the personal well-being with the study conduct will be addressed. The study was approved by the responsible ethics committee.

## Discussion

### Expected results of the study

This study will identify strengths, weaknesses and gaps of perinatal mental health care in Switzerland. Based on the results we will propose recommendations to optimize perinatal mental health care. We assume that our results are relevant internationally i.e. for health care contexts of other high income countries.

### Strengths and possible limitations

Our study will be strengthened by our cooperation with psychiatrists from the University of Bern, to ensure we can recruit and interview women from this highly vulnerable population without further burdening them during and after the interviews. Another strength of the study is that we include patients who were diagnosed based on ICD10 by a psychiatrist, while other studies rely on self-assessment of patients [21].

Generalizability of our study results will be limited to patients who received perinatal mental health care at the University of Bern, Switzerland. Generalizability will also be limited by responder bias, since we will include only former patients with PMD who are interested in our research topic. This purposive sample may also have its strengths, since these women are highly motivated to report their experiences and can provide us with competent statements.

### Importance and benefits of the study

The high and growing burden of mental illness in the perinatal period makes it necessary to reform health care [41, 42]. This applies especially for women with PMD because inadequate treatment can cause severe and long-lasting harm for affected women, their children and families [4, 43, 44] and high long-term costs for society [8]. So far, we do not know enough how PMD patients interact with mental health care, or which specific health care needs are associated with PMD. Therefore, this study will contribute to this highly relevant but under-researched field of reproductive health care.

By considering a broad range of the most frequent and most severe PMD the study will generate results which are relevant for a wide range of different PMD patients and across various health care settings. The wide interprofessional approach of the focus groups and the combined perspectives of users and providers of health and social care services will open up new perspectives of what is relevant for adequate perinatal mental health care. The comprehensive approach will identify barriers and facilitators that affect access to care, timeliness and adequacy of care. Our data will inform the design of innovative interdisciplinary models of care – including advanced practice roles for nurses and midwives. The data may inform the development of interventions and guidelines. The results and conclusion of the study will be relevant for health care research and practice in Switzerland and other high-income countries.

## Supplementary information

**Additional file 1: Table S1.** First draft of the semi-structured interview guide for individual interviews with women who have had perinatal mental disorders (MADRE, Switzerland 2019). **Table S2.** First draft of the socio-demographic survey for women who have had perinatal mental disorder and participate in the study (MADRE, Switzerland 2019). **Table S3.** First draft of the focus group interview guide (MADRE, Switzerland 2019). Interview questions are based on the style of Krueger and Casey (see [37]). **Table S4.** First draft of the socio-demographic survey for health and social care professionals who participate in the focus groups (MADRE, Switzerland 2019).

## Data Availability

Interview guides and socio-demographic questionnaires generated for this study protocol are included in Additional file 1.

## Abbreviations

BSI: Brief Symptom Inventory
ICD10: International Classification of Diseases
PMD: Perinatal mental disorder
