# Supplementary material for: Perinatal mental health care from the user and provider perspective: protocol for a qualitative study in Switzerland
Source: Reprod Health. 2020 Feb 17;17:26. doi: 10.1186/s12978-020-0882-7 (PMC7027089; doi:10.1186/s12978-020-0882-7)
Supplement: Supplementary file 1 — Additional file 1: Table S1. First draft of the semi-structured interview guide for individual interviews with women who have had perinatal mental disorders (MADRE, Switzerland 2019). Table S2. First draft of the socio-demographic survey for women who have had perinatal mental disorder and participate in the study (MADRE, Switzerland 2019). Table S3. First draft of the focus group interview guide (MADRE, Switzerland 2019). Interview questions are based on the style of Krueger and Casey (see [37]). Table S4. First draft of the socio-demographic survey for health and social care professionals who participate in the focus groups (MADRE, Switzerland 2019). [file 12978_2020_882_MOESM1_ESM.pdf]

Additional file 1

Journal: Reproductive Health

Article title: Perinatal mental health care from the user and provider perspective: protocol for a qualitative study in Switzerland

Authors:

Anke Berger[1*], Karin Schenk[1], Ankica Ging[2], Sebastian Walther[2,3], Eva Cignacco[1]

[1]Department of Health Professions, Bern University of Applied Sciences, Bern Switzerland

[2]University Hospital of Psychiatry, University of Bern, Bern, Switzerland;

[3]Translational Research Center, University Hospital of Psychiatry, University of Bern, Bern, Switzerland.

*Corresponding author

Supplementary Table 1: First draft of the semi-structured interview guide for individual interviews with women who have had perinatal mental disorders (MADRE, Switzerland 2019).

Questions of the semi-structured interview
Dear. ________, thank you for participating in the interview. During pregnancy / after the birth of your baby, you did not feel well.
**1. Could you tell me your experiences during that time?**
Optional:<br>• How did you feel as an expectant mother or as a mother? Did you have any specific concerns about the baby, your husband/partner, or your family?<br>• What were your main thoughts about the pregnancy or your baby after birth?
**2. Could you tell us about the circumstances of realizing that you became unwell?**
Optional:<br>• When did you notice that you became unwell?<br>• How did you recognize you were ill?<br>• What was it like to be ill? What kind of symptoms did you have?<br>• At what point did you recognize that you needed help?
**3. How did you seek help?**
Optional:<br>• Who did you talk to or seek help from?<br>• Were there any barriers to seeking help?<br>• How did you feel when you talked to a healthcare professional about your feelings? Did you think they understood?<br>• Were you comfortable talking to healthcare professional about your feelings? Were there any concerns or anxieties?
**4. After talking to a health care professional or seeking help, what happened?**
Optional:<br>• Which service/s were you referred to? What treatment did you receive?<br>• How did you feel and think about this referral? Did you have any concerns?<br>• How long did it take to be seen once you have been referred to a specialist?
**5. How did you experience the treatments or services?**
Optional:<br>• Which person, what kind of support or treatment helped you most?

- What would you have liked to be different related to the experienced healthcare provision?
- Are there any appointments?
- Do you think the healthcare professionals were competent?
- Did you experience an excessive demand of the healthcare professionals?

6. Now I would like to ask you some questions about the communication with healthcare professionals about your mental health: **Before feeling unwell or seeking help, how did healthcare professionals obtain information from you about your mental wellbeing?**

Optional:

- Did healthcare professionals ask you about your feelings/your mood?
- How was the communication about mental health with your healthcare professional?
- What aspects were helpful for the communication, what hindering?
- Did you feel confident about talking openly and honestly about your feelings and mood?
- Is there a reason why you didn't feel comfortable talking to your health professional about how you were feeling? Were you afraid of something?
- In general, how was the relationship with your healthcare professional?

7. **At the beginning of your pregnancy – before you became unwell – what did you think or know about perinatal mental illness?**

Optional:

- Did a healthcare professional inform you about perinatal mental illness?
- How did you receive the information about perinatal mental illness? (e.g., written information materials)
- Do you think it is important that women are informed about perinatal mental illness at the beginning of their pregnancy? Why?

8. **What advice would you give to a woman beginning her pregnancy who is also experiencing mental illness?**

9. **What advice would you give to a health care professional about caring for a woman who is also experiencing mental illness during pregnancy or after the baby is born?**

10. **What gaps, if any, do you feel there are in the mental health support given to women before, during and after pregnancy?**

11. **What strategies would be helpful to optimize maternal mental health care?**

12. **If you look back, what would you do differently today?**

13. **Is there anything else you would like to share?**

.

Supplementary Table 2: First draft of the socio-demographic survey for women who have had perinatal mental disorder and participate in the study (MADRE, Switzerland 2019).

Questionnaire for assessing individual data of the participants of the interview

Thank you for participation in the study. Before we start with the interview, we would appreciate it if you could complete the following questions about some demographical data.

All responses to this questionnaire and the following interview will be completely confidential.

1. How old are you?	________ years
2. How many children do you have?	________ children
3. How many months is the birth of your (last) baby back?	________ months
4. How is your marital status?	□ married<br>□ in a relationship (unmarried)<br>□ divorced / separated<br>□ single mother<br>□ other: ________
5. How is your level of education?	□ Secondary school<br>□ High school diploma<br>□ Professional honor diploma<br>□ Bachelor/Master diploma<br>□ Doctorate/PhD diploma<br>□ other: ________
6. Do you work actually?	□ No<br>□ Yes ____ %
7. What is your nationality?	________
8. Did you have a previous history of mental health problems?	□ No<br>□ Yes
9. What kind of mental health sevice did you receive?	________
10. Did or do you have any prescribed medication?	□ No<br>□ Yes
If yes, what kind of medication was or is prescribed?	________

Thank you for answering the questions!

Supplementary Table 3: First draft of the focus group interview guide (MADRE, Switzerland 2019). Interview questions are based on the style of Krueger and Casey (see reference list in the main text of the article

Type[1]	**Questions**
Opening question	I would like to start the interview with a short round of introduction. I would like to start the interview with a short round of introduction. Please tell us your name, your profession and where you practice.
	**1. Introductory questions**
Introductory question	Think back to your clinical practice and remember your experiences with women suffering from perinatal mental disorder during pregnancy or in the year after birth.<br>1.1 Please tell us your experiences in working with women suffering from perinatal mental disorder.<br>1.2 What made working with these mothers more challenging than working with mentally healthy mothers?<br>1.3 How did you handle that issue/situation?<br>1.4 Could you give us an example where maternal mental health care proceeded well / proceeded poorly?
	**2. Citation of a statement from an interviewed women**
Transition question	As a transition question, we will quote a woman with former PMD who related her experiences with perinatal mental health care and participated in one of our study interviews. Participants in the focus group are invited to share their thoughts about what she said.
	**3. Speaking with perinatal women about their mental wellbeing**
Key question	The following questions focus on speaking with perinatal women about their general mental wellbeing.<br>1.1 How do you gather information about perinatal women's mental health when you don't know if there are signs of a perinatal mental health disorder?<br>1.2 Are there some factors, like previous history of mental illness, or traumatic birth, that may influence how often you ask women about their emotional wellbeing?<br>1.3 Do you think and ask about social circumstances that could influence maternal mental health, like previous history of mental disorders, poor partner relationship, or a difficult mother-infant interaction?<br>1.4 Could you describe some barriers to discussing mental health and wellbeing with your patients?<br>1.5 Do the women speak about perinatal mental disorder on their own? What do they ask?<br>1.6 Do the women receive some psychoeducation about perinatal mental disorders?
	**4. Proceeding if there are some signs for a perinatal mental disorder**
Key questions	Now we would like to ask you what happens after a woman shows signs of a perinatal mental disorder.<br>3.1 Please tell us how you proceed when a woman is seeking help or when you recognize that a woman has some signs of a perinatal mental disorder.<br>3.2 Is the process difficult in any way?<br>3.3 Are there any differences between the procedures for women with pre-existing mental disorders and women with a first case of emerging perinatal mental disorder?<br>3.4 Does the specific diagnosis (for example, depression, anxiety disorder, psychosis) make a difference when you work with women with perinatal mental health disorder?

	3.5 In general, do you feel confident, competent, or uncertain when working with women with perinatal mental disorders?<br>3.6 What kind of resource or support would help you better serve affected women and their families?
	**5. Collaboration with different health care professionals/disciplines**
Key questions	Now we would like to ask you about your experiences with interdisciplinary collaboration in treating women with perinatal mental disorders.<br><br>4.1 What is your experience of collaboration with different health care professionals?<br>4.2 Can you describe support and information sharing between different disciplines of health care professionals?<br>4.3 Where do you detect gaps in health care provision for affected women in the perinatal phase?<br>4.4 How should collaboration between different services be organized and improved to prevent disconnected care?
	**6. Desired changes or improvements in maternal mental health care**
Key questions	Now we would like to ask you some general questions with a broad focus on all aspect of perinatal mental health care.<br><br>5.1 If you had the opportunity to change or improve something in caring for mothers with perinatal mental disorders during the perinatal period, what would it be?<br>5.2 How do you suggest the change(s) be implemented?<br>5.3 What do you think would most help mothers-to-be or mothers with mental health disorder during the perinatal period?
	**7. Education**
Key questions	Educating healthcare professionals is necessary to improve maternal mental health care and increase the confidence of healthcare professionals who working with women who have perinatal mental disorders.<br><br>6.1 What do you think about the education programs that cover maternal mental health care?<br>6.2 Do you have any recommendations to improve basic and ongoing education?
Ending question	*At the end of the focus group, the moderator repeats the purpose of the focus group and the study:*<br>“The aim of this focus group is to gather information about the experiences and perspectives of healthcare professionals and social workers who have cared for women with perinatal mental disorder.”<br>*After this, the moderator asks the final question:* “Is there anything we should have talked about, but didn’t?”

Supplementary Table 4: First draft of the socio-demographic survey for health and social care professionals who participate in the focus groups (MADRE, Switzerland 2019).

Questionnaire for assessing invidual data of the participants of the focus groups

Thank you for participating in one of the three focus groups. Before we start with the focus group interview, we would appreciate it if you could complete the following questions. Anonymity of the quesionnaire is assured. Your answers will only be used for describing the demographical data of the participants of each focus group.

**Gender**	□ Male<br>□ Female
**Age**	__________ Years
**Level of employment**	__________ %
**Occupational group**	□ Nurses<br>□ Midwives<br>□ Gynecologists<br>□ Primary care providers<br>□ Community services<br>□ Pediatricians<br>□ Social care providers<br>□ Psychiatrists<br>□ Psychologists
**Professional experience**	□ 2 - 5 years<br>□ 6 - 10 years<br>□ 11 - 15 years<br>□ 16 - 20 years<br>□ > 20 years

How many years of professional experience do you have in working with women with perinatal mental disorders?

__________ Years

Thank you for answering the questions!
